# Genome-Wide Investigation of the NAC Gene Family and Its Potential Association with the Secondary Cell Wall in Moso Bamboo

**DOI:** 10.3390/biom9100609

**Published:** 2019-10-14

**Authors:** Xuemeng Shan, Kebin Yang, Xiurong Xu, Chenglei Zhu, Zhimin Gao

**Affiliations:** 1 Institute of Gene Science for Bamboo and Rattan Resources, International Center for Bamboo and Rattan, Beijing 100102, China; shanxuemeng@icbr.ac.cn (X.S.); yangkebin@icbr.ac.cn (K.Y.); xuxiurong@icbr.ac.cn (X.X.); zhuchenglei@icbr.ac.cn (C.Z.); 2 National State Forestry and Grassland Administration/Beijing Key Open Laboratory on the Science and Technology of Bamboo and Rattan, Beijing 100102, China

**Keywords:** moso bamboo, NAC identification, expression analysis, secondary cell wall

## Abstract

NAC (NAM, ATAF, and CUC) transcription factors (TFs) are implicated in the transcriptional regulation of diverse processes and have been characterized in a number of plant species. However, NAC TFs are still not well understood in bamboo, especially their potential association with the secondary cell wall (SCW). Here, 94 *PeNAC*s were identified and characterized in moso bamboo (*Phyllostachys edulis*). Based on their gene structures and conserved motifs, the PeNACs were divided into 11 groups according to their homologs in *Arabidopsis*. *PeNAC*s were expressed variously in different tissues of moso bamboo, suggesting their functional diversity. Fifteen *PeNAC*s associated with the SCW were selected for co-expression analysis and validation. It was predicted that 396 genes were co-expressed with the 15 *PeNAC*s, in which 16 and 55 genes were involved in the lignin catabolic process and cellulose biosynthetic process respectively. As the degree of lignification in the growing bamboo shoots increased, all 15 *PeNAC*s were upregulated with a trend of rising first and then decreasing except *PeNAC37*, which increased continuously. These results indicated that these *PeNAC*s might play important roles in SCW biosynthesis and lignification in bamboo shoots. Seven of 15 *PeNAC*s had been found positively co-expressed with seven *PeMYB*s, and they had similar expression patterns with those of the *PeMYB*s in bamboo shoots. The targeted sites of miR164 were found in 16 *PeNAC*s, of which three *PeNAC*s associated with SCW were validated to have an opposite expression trend to that of miR164 in growing bamboo shoots. In addition, three *PeNAC*s were selected and verified to have self-activation activities. These results provide comprehensive information of the *NAC* gene family in moso bamboo, which will be helpful for further functional studies of *PeNAC*s to reveal the molecular regulatory mechanisms of bamboo wood property.

## 1. Introduction

With the improvement in living standards, the demand for timber is rapidly growing, and timber supply is decreasing greatly, resulting in a need for alternative resources [1]. The development and utilization of bamboo resources can effectively alleviate the problems of restricted timber supply and decreasing timber quantity. Bamboo has a fast growth rate, high yield, and good material properties, such as high strength, good toughness, and high hardness compared with wood [2,3]. Bamboo is considered one of the important biomass energy sources. The formation and thickness of the secondary cell wall (SCW) play important roles during plant growth, producing a lignified texture and forming wood. The lignified SCW is the most environmentally friendly and cost-effective renewable resource that can improve lignocellulosic biomass [4]. Currently, the SCW of plant cells has received much attention [5,6]. Transcriptional regulatory pathways play a pivotal role in SCW biosynthesis. Many findings have revealed the regulation of SCW biosynthesis at the molecular level, indicating that plants have specific transcriptional switches regulating SCW biosynthesis [7,8,9,10]. Many transcription factors (TFs) are reported to be involved in the formation of the SCW by regulating the synthesis of SCW components such as cellulose, hemicellulose, and lignin [11].

The NAC (NAM, ATAF, and CUC) gene family is considered to be one of the largest families of plant-specific TFs, and its members play significant roles in diverse biological processes, including senescence [12,13], hormone signaling [14], and SCW biosynthesis [15]. In the formation of the SCW, three NAC TFs (NST1, NST2, and NST3/SND1) are defined as master regulators for their functions in regulating all three components, i.e., cellulose, hemicellulose, and lignin biosynthesis in xylary fibers [16]. The secondary wall thickening of the anther endothecium is controlled by *NST1* and *NST2* in *Arabidopsis* [17]. Many other *NAC* genes have been shown to have important roles in SCW biosynthesis, such as VASCULAR-RELATED NAC-DOMAIN1-7 (*VND1*-*VND7*) [18,19,20]. Overexpression of *VND* genes upregulates the expression of genes associated with the SCW and increases cell wall thickness [18]. *GhFSN1,* a cotton NAC gene, has been identified as a positive regulator participating in the control of SCW biosynthesis and modification of fibers [21]. In addition to NAC TFs, MYB TFs, and many other TFs are members of the gene regulatory network controlling SCW biosynthesis [22]. Co-expression relationships have been found between fiber-associated *NAC* genes and SCW biosynthetic genes in cotton [23]. The functions of the *NAC* genes associated with SCW are relatively conserved throughout the plant kingdom [21,24,25]. However, the functions of NACs associated with SCW in bamboo remain unclear thus far.

Bamboo is one of the most important non-timber forest resources cultivated on marginal land for biobased products and is considered an emerging and important lignocellulosic biomass energy source [26]. Moso bamboo (*Phyllostachys edulis*) belongs to the subfamily Bambusoideae of the Poaceae family, along with most of the ecological and economic bamboo types in the world, it can be used to produce timber, artwork, paper, and food (young bamboo shoots) [3,27]. Moso bamboo has a quick growth rate with a maximum height of approximately 20 m, which is reached in one and a half months [28], and its degree of lignification continuously increases with increasing height [29]. The SCW becomes thickened and lignified during rapid growth, playing an important role in improving the properties and application of bamboo [30]. Many NAC TFs associated with SCW have been identified in *Arabidopsis* [31], rice [32], foxtail millet [33], and cotton [23], and the homologous genes differ in gene structure and function among different species. However, little is known about NAC TFs related to SCW in moso bamboo. The completion of the moso bamboo draft genome [28] and the bamboo genome database (BambooGDB) [34] facilitate the exploration of the specific structural and functional characteristics of moso bamboo *NAC* genes for further study. The purpose of this study is to gain a comprehensive understanding of NAC TFs in moso bamboo, which will be helpful to reveal the molecular mechanisms of NAC TFs involved in the biosynthesis of SCW.

## 2. Results

### 2.1. Identification of NACs in Moso Bamboo

To identify *NAC* genes in moso bamboo, a total of 125 candidates were downloaded from the PlantTFDB. Furthermore, BLASTX and BLASTP were used to examine the NAM domain, and the Clustal W program was used to compare the presence of the NAM domain in the sequences. Finally, 94 NAC proteins with complete conserved domain were obtained through a comprehensive analysis. For convenience, the prefix “Pe”, which stands for *Phyllostachys edulis*, was placed before the gene family name (NAC), and the 94 *NAC* genes were named *PeNAC1*-*PeNAC94* sequentially. The characteristic parameters of the predicted proteins encoded by the *PeNAC*s are shown in Table S1, including protein length, molecular weight, theoretical isoelectric point (pI), and subcellular localization. The lengths of the PeNACs ranged from 217 aa (PeNAC49) to 694 aa (PeNAC41), with an average of 376.7 aa, their molecular weights varied from 25201.17 Da (PeNAC49) to 76916.46 Da (PeNAC41), and their pI values were from 4.55 (PeNAC79) to 10.85 (PeNAC15). In addition, the prediction of subcellular localization revealed that all 94 PeNACs were localized in the nucleus.

### 2.2. Gene Structure and Conserved Motif Analyses of PeNACs

**Figure 1:**
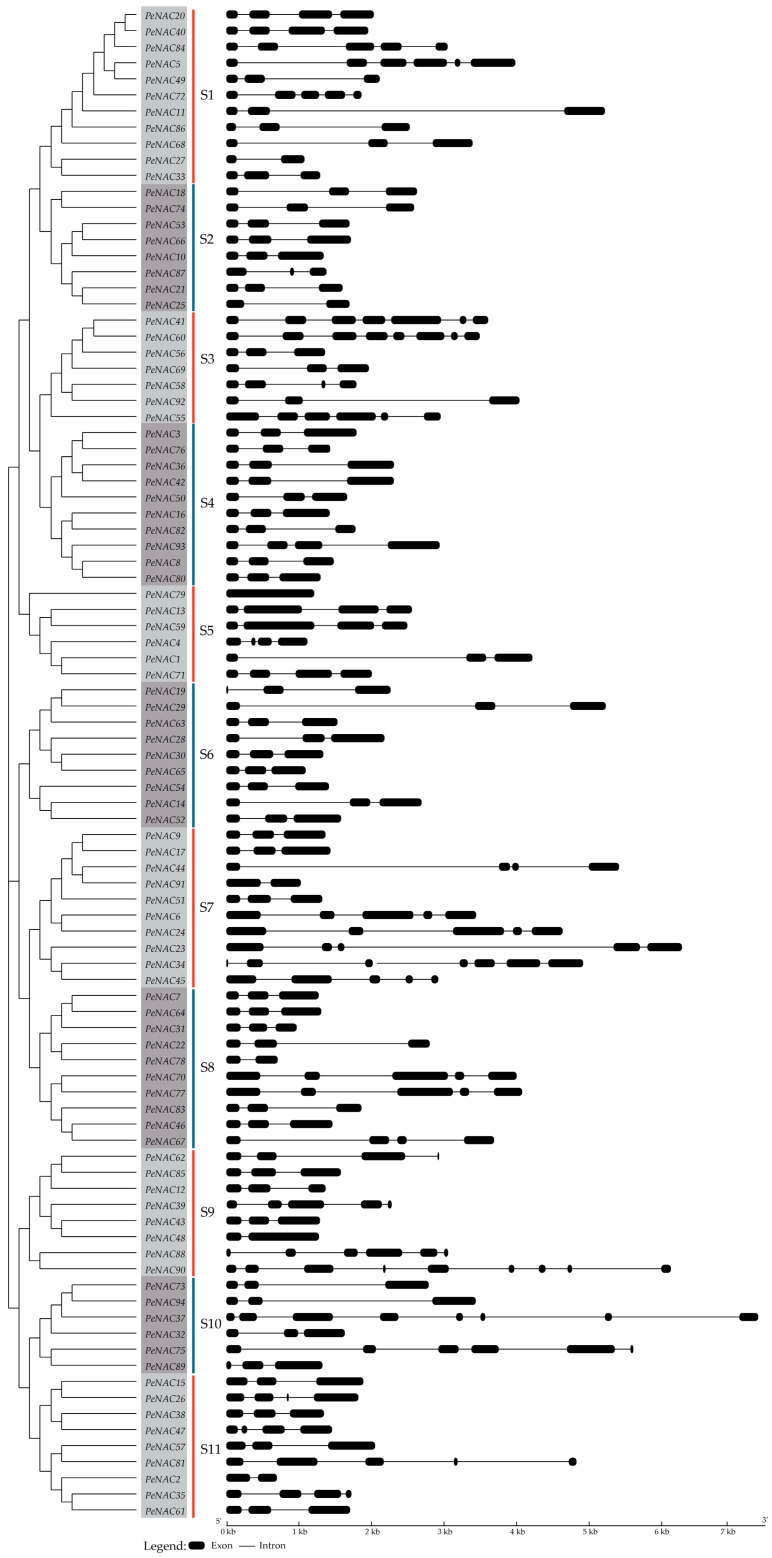
Exon–intron structures of *PeNAC*s in moso bamboo. The black boxes represented exons and spaces between the black boxes corresponded to introns.

**Figure 2:**
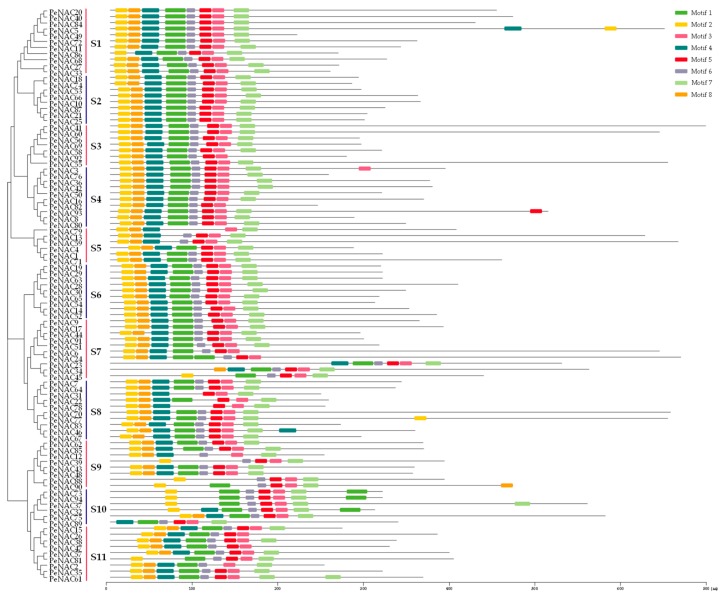
Schematic representation of the conserved motifs in PeNACs. Motifs were identified using the MEME online tool. Each motif was indicated by different colored blocks (motif 1–motif 8). The position and length of each colored box represented the actual motif size.

To better understand the diversity and similarity of the *PeNAC*s, the exon–intron organization and conserved motifs were further analyzed. The results showed that all the PeNACs were clustered into 11 subgroups, named S1–S11, based on the phylogenetic tree (Figure 1 and Figure 2). The number of members in each subgroup ranged from 6 (S5 and S10) to 11 (S1). Gene structure analysis showed that the numbers and positions of introns in the *PeNAC*s were diverse, and the numbers ranged from 0 (*PeNAC79*) to 8 (*PeNAC90*) (Figure 1). Among them, the number of *PeNAC*s with two introns was the most with 54 genes, followed by 14 *PeNAC*s with three introns and 10 *PeNAC*s with four introns. Most *PeNAC*s in the same subgroups had similar exon–intron structures. For example, the *PeNAC*s with the maximum number of introns were clustered in S9, including eight members with eight introns. The intron numbers in each subgroup supported their close phylogenetic relationships and subgroup classifications. However, there were some exceptions: *PeNAC79*, with no introns, was clustered in S5, which included five *PeNAC*s with three introns and *PeNAC1* with two introns.

As shown in Figure 2, the diversity of motif compositions in the PeNACs was analyzed using MEME. A total of eight conserved motifs (motif 1–motif 8) were identified, and a schematic overview of the identified motifs was provided. Further analysis showed most of the conserved motifs were found to be located in the N-terminal region of the NAC domain, indicating that these conserved motifs were important to the functions of the PeNACs. Additionally, similar motif compositions were found to cluster in the same subgroup. For example, the members of S10 mainly contained six motifs (motif 1, 2, 3, 5, 6, and 7), while those in S1 held eight motifs (motif 1, 2, 3, 4, 5, 6, 7, and 8), suggesting that PeNACs in the same subgroup may have similar functions.

### 2.3. Phylogenetic Analysis and Classification of PeNACs

**Figure 3:**
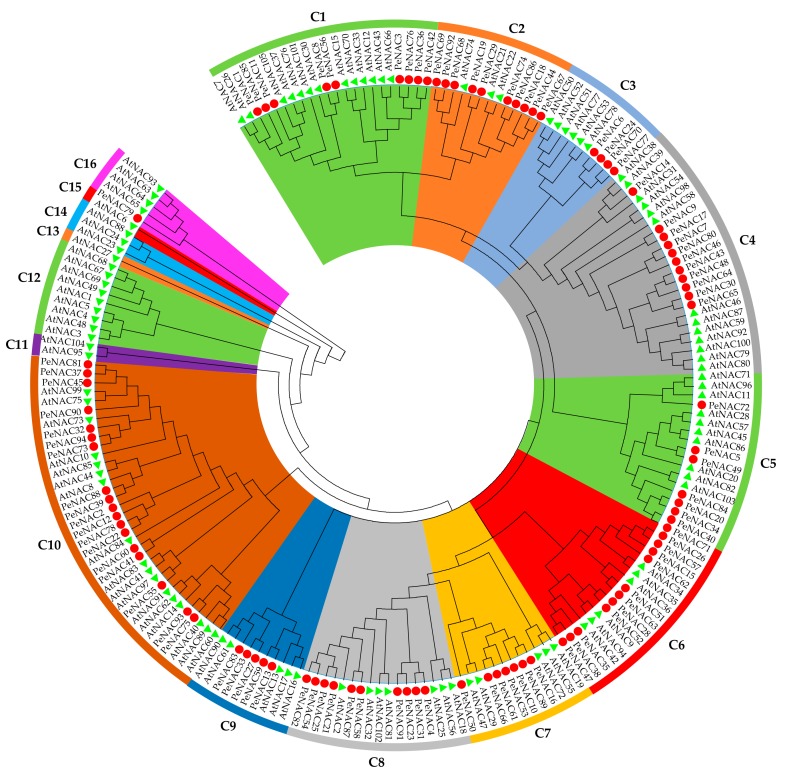
Phylogenetic tree based on the proteins of moso bamboo and *Arabidopsis*. The tree was constructed using the neighbor-joining (N–J) method with 1000 bootstrap replicates as implemented in MEGA6.0. The names of clade were shown outside of the circle. Geometric figures of different colors and shapes were used to mark the NAC members from different species.

To reveal the phylogenetic relationships of NAC family members from moso bamboo and *Arabidopsis*, a neighbor-joining (N-J) phylogenetic tree was constructed from the amino acid sequences via multiple alignment. Detailed information on the NACs from moso bamboo and *Arabidopsis* was shown in Table S2, and the results indicated that all the NACs had been clustered into 16 clades (C1–C16). The 94 PeNACs were distributed in 11 clades (C1–C10 and C15), and each clade contained 1–18 members (Figure 3 and Figure S2). The phylogenetic analysis also showed that there were some closely related NACs between moso bamboo and *Arabidopsis* (such as AtNAC97 and PeNAC55; AtNAC36, PeNAC51, and PeNAC63; AtNAC38, AtNAC39, and PeNAC14), suggesting that these PeNACs may have similar functions to those of their homologous AtNACs in *Arabidopsis*.

### 2.4. Putative Functions of PeNACs in Moso Bamboo

The functions of NAC TFs in *Arabidopsis* have been well studied [31]. Based on the N-J phylogenetic tree constructed using 94 PeNACs and 105 AtNACs, homologous NACs that clustered together were speculated to have similar functions. Therefore, the functions of the PeNACs were predicted by comparing them with those of the AtNACs (Figure 3). The results showed that the NAC members in these two species were divided into 16 clades (C1–C16), including 11 common clades shared by the two species and five species-specific clades in *Arabidopsis*. There were 65 PeNACs belonging to six functionally annotated clades and 29 belonging to five functionally unknown clades. The 65 PeNACs were divided into five functional classes. Class I, including two clades (Clade1 and Clade10), was responsible for SCW formation by regulating the biosynthesis and deposition of lignin. Class II, III, and IV all contained one clade, the function of Class II (Clade2) was related to sequence-specific DNA binding, Class III (Clade4) played important roles in the formation of the apical meristem and the establishment of organ boundaries, and Class IV (Clade10) was involved in plant regulatory and signal transduction reactions. Class V, including two clades (Clade7 and Clade8) might be responsible for plant aging and abiotic stress, such as high salt and drought stresses. These results suggested that PeNACs have diverse functions and may have significant roles in the growth and development of moso bamboo.

### 2.5. Expression Patterns of PeNACs Based on Transcriptome Data of Moso Bamboo

**Figure 4:**
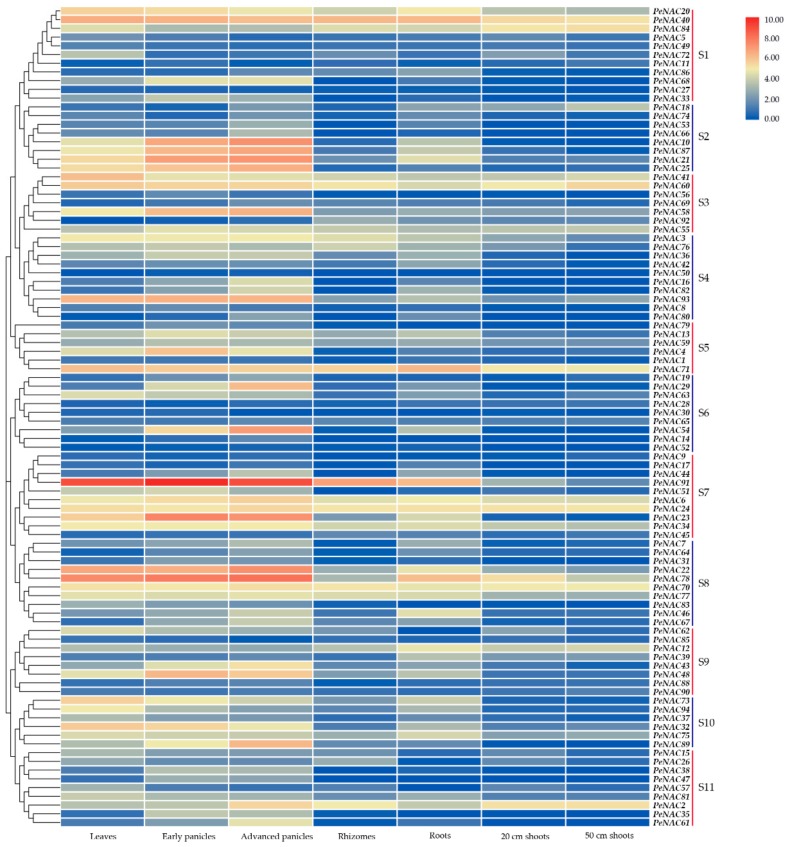
Expression patterns of *PeNAC*s in different tissues/organs of moso bamboo based on the transcriptome data. Heatmap represented for the expression of *PeNAC*s in leaves, early panicles, advanced panicles, rhizomes, roots, 20 cm shoots, and 50 cm shoots. Color scale represented log_2_ expression values, with the color from blue to red indicating low to high expression abundance.

To better understand the roles of the *PeNAC*s, transcriptome data sets from different tissues/organs, including the leaves, panicles, rhizomes, roots, and shoots of moso bamboo, were used to explore the expression patterns of the 94 *PeNAC*s. The results showed that they had different expression patterns in different tissues, and most of them showed significant tissue specificity. The expression of all 94 *PeNAC*s was detected in at least two tissues, and 63 *PeNAC*s were detected in all seven tissues, with transcript abundances ranging from 0.0283028 to 961.932 (reads per kilobase per million mapped reads—RPKM). In addition, some *PeNAC*s had high expression levels in specific tissues. For example, *PeNAC*s belonging to S7, S8, and S9 mostly showed higher expression levels in the leaves and panicles but relatively lower levels in the other four tissues, especially in shoots. Interestingly, 12 *PeNAC*s (*PeNAC20*, *PeNAC40,* and *PeNAC84* in S1, *PeNAC41* and *PeNAC60* in S3, *PeNAC71* in S5, *PeNAC6*, *PeNAC24,* and *PeNAC34* in S7, *PeNAC70* and *PeNAC78* in S8, and *PeNAC2* in S11) showed high expression in almost all the tested tissues. Meanwhile, the *PeNAC*s belonging to S3 showed high expression levels, and those belonging to S6 had low expression in all seven tissues (Figure 4).

### 2.6. Expression Correlation and Co-Expression Networks

**Figure 5:**
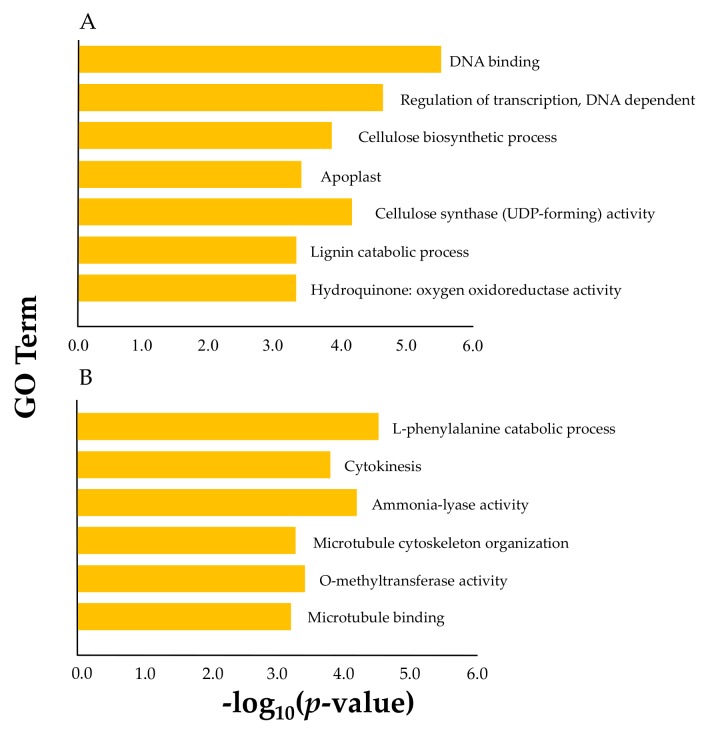
Gene Ontology (GO) enrichment analysis of the genes co-expressed with 15 *PeNAC*s associated with SCW. (**A**): GO term of the co-expression genes with *PeNAC*s of Clade1; (**B**): GO term of the co-expression genes with *PeNAC*s of Clade1. The *x*-axis was log_10_ (*p*-value) and *y*-axis was the GO term.

Fifteen *PeNAC*s associated with SCW belonging to Clade1 and Clade10 were further analyzed to reveal their co-expressed genes. Based on the 78 transcriptional datasets [35], we found that the numbers of genes co-expressed with the *PeNAC*s of Clade1 and Clade10 were 236 and 160, respectively. The functions and related biological pathways of the co-expressed genes were enriched in two SCW biosynthesis-related biological processes, lignin catabolic process and cellulose biosynthetic process, according to the subsequent Gene Ontology (GO) analysis. Comparing the co-expressed genes of the *PeNAC*s in Clade1 to those in Clade1, the former appeared more closely related to the function of SCW biosynthesis (Figure 5). This result suggested that these *PeNAC*s might be involved in lignification by regulating SCW synthesis.

**Figure 6:**
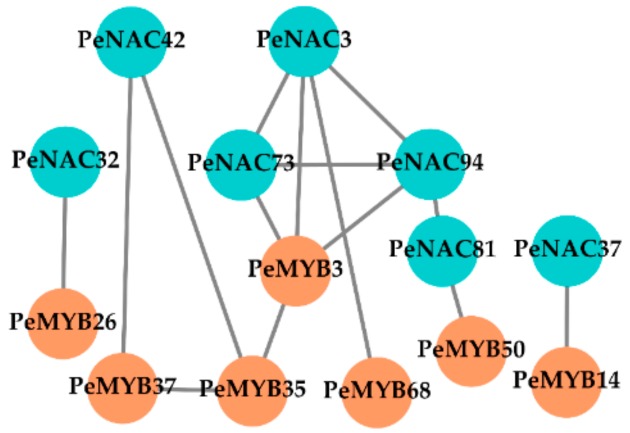
Positive co-expression network of *PeNAC*s and *PeMYB*s. The green and orange circles represent *PeNAC*s and *PeMYB*s respectively.

**Figure 7:**
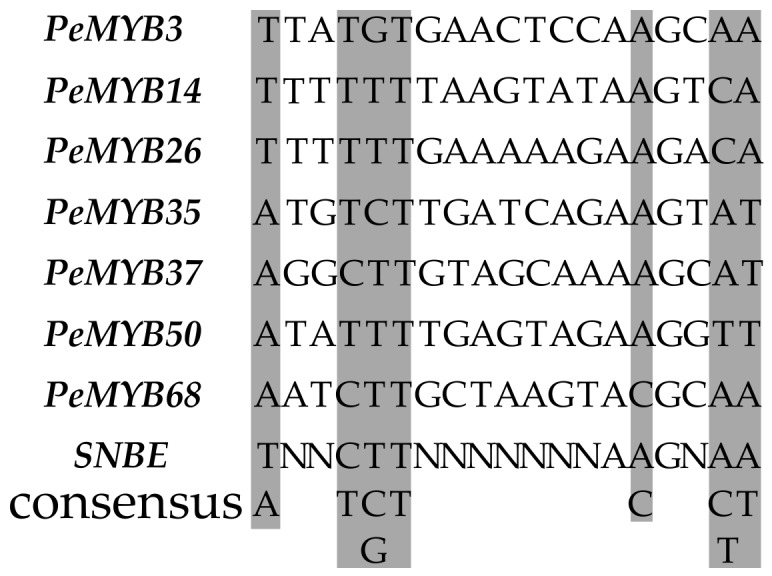
Diagram of the secondary wall NAC binding element (SNBE) sequences in the 2.0-kb promoters of *PeMYB*s. The consensus nucleotides in the SNBEs were shaded and the consensus SNBE sequence was shown at the bottom.

In addition, seven *PeMYB*s were found to be positively co-expressed with seven of the 15 *PeNAC*s (Figure 6), and their 2.0-kb promoters all had secondary wall NAC binding element (SNBE) that consisted of a 19-bp imperfect palindromic consensus sequence, (T/A)NN(C/T)(T/C/G)TNNNNNNNA(A/C)GN(A/C/T)(A/T) [36] (Figure 7), suggesting that the *PeNAC*s could regulate the *PeMYB*s through binding the SNBE element in their promoters.

### 2.7. Prediction of miRNA Target Sites in PeNACs

Many microRNAs (miRNAs) have been reported to be involved in SCW biosynthesis [37,38,39]. MiRNAs can negatively regulate target genes by partially pairing to the corresponding mRNA and facilitating its cleavage [40]. The online website psRNATarget was used to identify the target sites of miRNAs in *PeNAC*s. Due to the close relationship between rice and moso bamboo [28], the miRNA sequences that may regulate *PeNAC*s were analyzed using a rice miRNA library as a reference. The results showed that a total of 315 miRNA types with 563 target sites were predicted in the 94 *PeNAC*s, of which *PeNAC5* had the most miRNA types (91) and *PeNAC25* contained the fewest types (3) (Table S3 and Table S4). These results suggested that each gene might be regulated by multiple miRNAs. In addition, some *PeNAC*s contained the same miRNA target sites. For example, the putative target site of miR164 was found in 16 *PeNAC*s, and that of miR397 was found in 11 *PeNAC*s. There were 15 *PeNAC*s associated with SCW synthesis, among which three *PeNAC*s (*PeNAC36*, *PeNAC42,* and *PeNAC45*) were putative targets of miR164. These results suggested that not all genes with similar functions were regulated by the same miRNA, and the same miRNA could regulate multiple genes with different functions.

### 2.8. Expression Changes in Different-Height Shoots of Moso Bamboo

**Figure 8:**
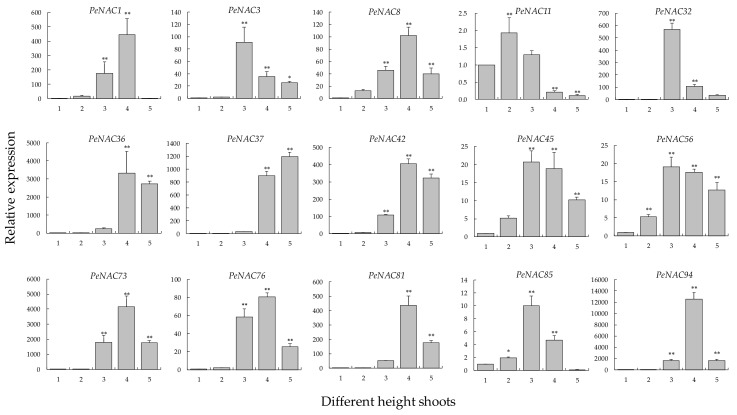
Expression analysis of 15 *PeNAC*s in different height shoots using qRT-PCR. *PeNTB* was used as the reference gene. Average and error bars represented standard deviation of three biological replicates. Asterisks indicated significant difference compared to the transcription level of control groups (* *p* < 0.05, and ** *p* < 0.01). 1: 1.0 m shoots; 2: 2.0 m shoots; 3: 4.0 m shoots; 4: 6.0 m shoots; and 5: 8.0 m shoots.

The degree of lignification continuously increases with increasing height. To explore the functions of the 15 *PeNAC*s associated with SCW, their expression patterns in moso bamboo shoots of different heights were investigated using qRT-PCR with *NTB* (nucleotide tract-binding protein gene) [41] as the reference gene. The results showed that with increasing bamboo shoot height, the expression of 15 *PeNAC*s was upregulated, with a similar trend of first increasing and then decreasing, except for *PeNAC37*, which showed a continuously rising trend (Figure 8). The expression of *PeNAC37* was upregulated more than 1000 times in 8.0 m shoots compared to that in 1.0 m shoots. Moreover, there were some differences in the expression patterns: The expression of five *PeNAC*s (*PeNAC3*, *PeNAC32*, *PeNAC45*, *PeNAC56,* and *PeNAC85*) peaked in 4.0 m shoots, that of eight *PeNAC*s (*PeNAC1*, *PeNAC8*, *PeNAC36*, *PeNAC42*, *PeNAC73*, *PeNAC76*, *PeNAC81,* and *PeNAC94*) in 6.0 m shoots, and that of *PeNAC11* in 2.0 m shoots. Except for *PeNAC11*, the maximum expression level of the *PeNAC*s was more than two times that in 1.0 m shoots, in which the maximum was that of *PeNAC94*, with more than 12,565 times.

**Figure 9:**
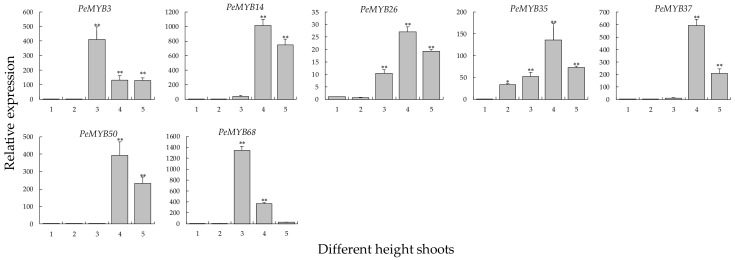
Expression analysis of 7 *PeMYB*s in different height shoots using qRT-PCR. *PeNTB* was used as the reference gene. Average and error bars represented standard deviation of three biological replicates. Asterisks indicated significant difference compared to the transcription level of control groups (* *p* <0.05, and ** *p* <0.01). 1: 1.0 m shoots; 2: 2.0 m shoots; 3: 4.0 m shoots; 4: 6.0 m shoots; and 5: 8.0 m shoots.

Interestingly, there was also a certain difference in the degree of decline after the peak. The expression of nine *PeNAC*s (*PeNAC1*, *PeNAC3*, *PeNAC8*, *PeNAC32*, *PeNAC73*, *PeNAC76*, *PeNAC81*, *PeNAC85,* and *PeNAC94*) showed a significant decrease (more than 70% of the maximum), and that of five *PeNAC*s (*PeNAC11*, *PeNAC36*, *PeNAC42*, *PeNAC45,* and *PeNAC56*) was only approximately 20%. Moreover, the upregulated expression patterns of three *PeNAC*s (*PeNAC36*, *PeNAC42,* and *PeNAC45*) were opposite to that of miR164 (Figure S1), indicating that miR164 might be involved in regulating these *PeNAC*s during the growth of bamboo shoots. Additionally, the qRT-PCR results of *PeMYB*s showed that their expression patterns were similar to those of the co-expressed *PeNAC*s with first increasing and then decreasing in different-height shoots of moso bamboo (Figure 9).

**Figure 10:**
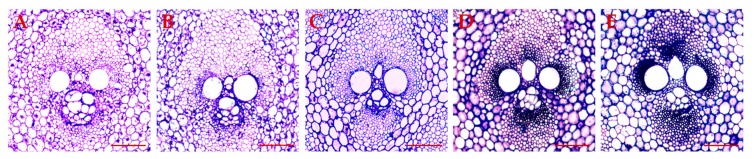
Transverse sections of vascular bundle in bamboo shoots with different heights. (**A**): 1.0 m shoots; (**B**): 2. 0 m shoots; (**C**): 4.0 m shoots; (**D**): 6.0 m shoots; and (**E**): 8.0 m. Scale bar: 100 μm.

Generally, the degree of lignification in bamboo shoots increases continuously with shoot growth [29]. Histological analysis showed that the number of stained cells in the vascular bundle increased and the SCWs thickened with increasing bamboo shoot height, indicating that the degree of lignification increased (Figure 10).

The expression of the 15 *PeNAC*s was all upregulated, consistent with the increasing degree of lignification, suggesting that they had potential associations with SCW biosynthesis. In addition, the *PeNAC*s exhibited diverse expression patterns in the developing bamboo shoots, which indicated that they might play different important roles in the shoot growth of moso bamboo.

### 2.9. Transcriptional Activity Assay

**Figure 11:**
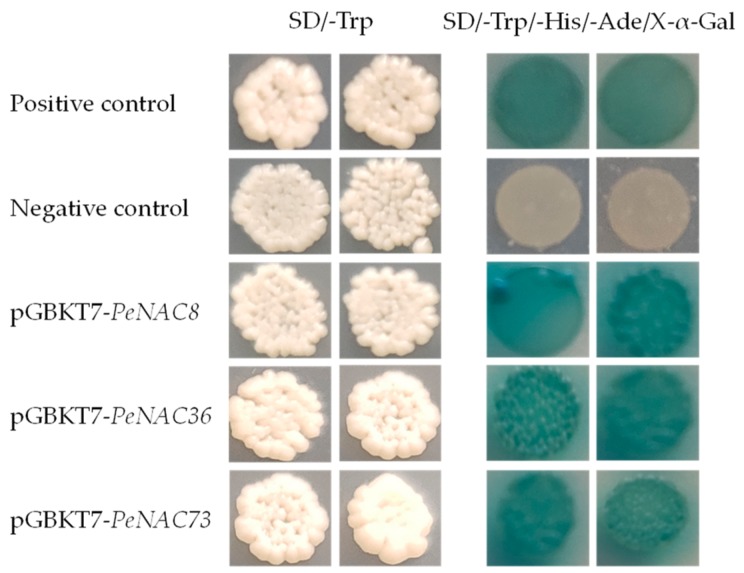
Assay of *PeNAC*s transcriptional activation activity in yeast. The positive constructs, negative constructs, and *PeNAC*s fused constructs were transformed into AH109 strains respectively, and successively incubated in SD/-Trp media and SD/-Trp/-His/-Ade supplemented with X-α-Gal.

The ORF sequences of *PeNAC8*, *PeNAC36,* and *PeNAC73* were isolated and verified by sequencing. Yeast expression vectors (pGBKT7-*PeNAC8*, pGBKT7-*PeNAC36,* and pGBKT7-*PeNAC73*) were constructed and transformed into the AH109 yeast strain. The results showed that all the plasmids could grow and exhibit visible white colonies on SD/-Trp, while on the SD/-Trp/-His/-Ade/X-α-Gal selective medium, only the positive control and the pGBKT7-*PeNAC*s yeast cells could grow well and turned blue. In contrast, the negative control could not grow on the selective medium (Figure 11). These results suggested that *PeNAC8*, *PeNAC36*, and *PeNAC73* all activated the expression of the reporter gene *lac*Z, indicating that they might be transcriptional activators for the regulation of SCW biosynthesis.

## 3. Discussion

NAC TFs have been confirmed to play important roles in many biological processes, including the formation and maintenance of the shoot apical meristem [42], hormone signaling [43], cell division [44], and, notably, the regulation of SCW synthesis [45]. However, there is still no detailed information available on the NAC family in moso bamboo, especially the roles of its members in SCW formation and deposition, which is associated with the quality of bamboo timber. To explore the NAC family in moso bamboo, the gene structure, conserved motifs, evolutionary relationships, functional prediction, tissue-specific expression, and transcriptional activity were analyzed in this study, and the results would facilitate an understanding of the regulatory mechanisms of NAC TFs in moso bamboo.

### 3.1. Conservation and Diversity of PeNACs

According to BLAST and Clustal W, all the identified NACs contained the NAM domain. Numbers of NAC family members are diverse in different plants. In this study, 94 *PeNAC*s were identified in moso bamboo, fewer than in *Arabidopsis* (125) [31], rice (151) [32], and tobacco (152) [46]. There were extensive variations in gene length, predicted protein molecular weight, and protein isoelectric point, whereas the gene structures were relatively conserved in the NAC gene family [23]. The NAC members in the same subgroups might have similar exon/intron structures and positions, indicating that these members were closely related and might have similar functions, consistent with previous studies [47,48]. However, there were a few differences between the members in the same subgroup, such as the size, relative location and number of exons. For example, *PeNAC81* in S11 had five exons, while the other S11 members had three to four exons, and its length was significantly longer than those of the other members. This result might be explained by the splicing or insertion of gene fragments during the evolutionary process, resulting in a new gene function [49,50]. In addition, motif detection confirmed that most of the NAC proteins in the same subgroup had the same motifs, which was evidence that these proteins have conserved functions. This was consistent with the statement that the functions of SCW-related NACs may be well conserved across the plant kingdom [21,25].

### 3.2. Homologous Genes Indicating Similar Functions

Semantic similarity between genes derived from GO hierarchy and annotations has been applied to predict gene function [51]. Based on a previous study, moso bamboo and rice have a close evolutionary relationship [28], but most of the *NAC* genes in rice have not been functionally verified. However, the *NAC* genes in *Arabidopsis* have been functionally well studied [31]; thus, we predicted the functions of the PeNACs according to those of *Arabidopsis*. As the phylogenetic tree shown, the NAC TFs of moso bamboo and *Arabidopsis* were clustered into 16 clades. According to the homology of AtNACs, the functions of the PeNACs were predicted, which indicated that they had functional diversity in the same clade. For example, AtNAC31, AtNAC92, and AtNAC98 belonged to C4, which is related not only to plant senescence and the formation of the apical meristem but also to the establishment of organ boundaries [52,53]. Interestingly, these results showed that PeNACs with similar functions were not clustered in one clade. For example, the PeNACs associated with SCW formation and lignin synthesis were clustered separately in C1 and C10. Simultaneously, there were some clades that had no functional annotations because there was no relevant research in *Arabidopsis*, and these clades require further verification.

### 3.3. Diverse Expression Patterns of PeNACs

Gene expression is one of the important manifestations of biological function. To further verify the tissue-specific expression of *PeNAC*s, we collected transcriptome datasets of moso bamboo [28], and the expression patterns of *PeNAC*s were analyzed in detail. We found that the *PeNAC*s belonging to S3 had high expression levels in all seven tissues, indicating that they might have an important role in the growth and development of moso bamboo. In contrast, the expression of *PeNAC*s in S6 was very low in all seven detected tissues, possibly because they might play roles in other undetected tissues. Interestingly, some *PeNAC*s mostly had high expression levels in the leaves and panicles, yet they showed low expression in early-stage moso bamboo shoots of 20 cm and 50 cm, suggesting they were mainly involved in cell differentiation and elongation rather than in the lignification process. The expression of 15 *PeNAC*s related to SCW synthesis was verified in the basal parts of the 13^th^ internode of moso bamboo shoots with different heights. The results showed that most of the *PeNAC*s had similar expression changes when compared with that in 1.0 m bamboo shoots, suggesting the conservation of their functions in regulating SCW biosynthesis, which is consistent with a previous study showing that as the height of the shoots increased, the degree of lignification increased [11]. Furthermore, the results of the histological analysis supported this conclusion.

### 3.4. Complexity of SCW Biosynthesis

Previous studies have shown that plants have specific transcriptional switches that regulate SCW biosynthesis, and these switches are TFs of the NAC and MYB families [9,10]. The plant-specific group of NAC TFs was considered to function as the first-layer master switch. In this study, the comprehensive expression analysis indicated that 15 *PeNAC*s were candidates involved in SCW biosynthesis, which were all upregulated with increasing bamboo shoot height. Moreover, *PeNAC37* was the only one with a continuously increasing expression trend; those of the other 14 *PeNAC*s tended to decrease after reaching a peak in the tested bamboo shoots, and the peak values of different *PeNAC*s were not in shoots of the same height, indicating that they functioned differently in time and space. The expression of miR164 had an opposite trend to those of three *PeNAC*s associated with SCW formation and lignin synthesis, consistent with the negative expression correlation between miRNAs and their target genes [54], indicating that there might be a miRNA-NAC regulatory model involved in the regulation of SCW formation in moso bamboo.

Some reports have implicated several MYB TFs as secondary master regulators of SCW formation [10,55], a NAC-MYB-based transcriptional regulation of SCW biosynthesis has been proposed, and the downstream structural genes involved in SCW formation have been reviewed [25,56]. In this study, seven *PeMYB*s that contained NAC binding element in their promoters were positively co-expressed with seven *PeNAC*s, and they had similar expression patterns to those of the co-expressed *PeNAC*s in different-height shoots of moso bamboo. It indicated that the *PeNAC*s could upregulate the expression of the *PeMYB*s, which was consistent with previous studies [25,57,58]. What is more, PeMYB3 was homologous to AtMYB46 and AtMYB83 clustered closely in the phylogenetic tree (30), which indicated that PeMYB3 is a potential second-layer master switch similar to AtMYB46 and AtMYB83 in the SCW formation [59,60,61]. These results suggested that the regulation of NAC-MYB in the synthesis of SCW was a progressive regulatory process. In addition, a total of 396 genes were predicted to be involved in the co-expression networks with the 15 *PeNAC*s, suggesting that the regulation of SCW biosynthesis in moso bamboo might involve an intricate network of TFs, structural genes, and miRNAs.

It has been reported that miRNAs are potential regulators of *NAC*s that in turn influence the *MYB*s [25,40]. In this study, miRNAs inhibit the RNA translation of *PeNAC*s, which result in the lower content of *PeNAC*s. Furthermore, less *PeNAC*s bind to the SNBE elements, which lead to lower expression of *PeMYB*s within the complex transcriptional regulation network of SCW biosynthesis process. However, a lot of research works are needed to reveal the regulatory mechanisms of this network involved in bamboo wood property.

## 4. Materials and Methods

### 4.1. Identification and Nomenclature of PeNACs

To gain a comprehensive and non-redundant list of proteins containing the NAM domain in moso bamboo, the NAC protein sequences were downloaded from the PlantTFDB (http://planttfdb.cbi.pku.edu.cn/index.php) [62]. To identify the members of the NAC family in moso bamboo, we performed BLASTX and BLASTP to examine the NAM domain and then used the Clustal W program to compare the proteins selected for amino acid sequence alignment to determine whether they contain the NAM domain. Finally, we manually checked these proteins to ensure that all had NAM protein domain. In combination with the previous alignments, proteins that did not contain this domain were deleted from the analysis. We regarded proteins with the NAM domain as predicted NAC proteins.

### 4.2. Gene Structure, Protein Characterization, and Sequence Analyses

The basic characteristics of the potential NAC TF members in moso bamboo were further analyzed, including the predicted proteins and their physicochemical parameters. The molecular weights (MWs) and theoretical isoelectric points (pIs) of the NAC proteins were predicted using ProtParam (http://web.expasy.org/protparam/), and the online software Plant-mPLoc (http://www.csbio.sjtu.edu.cn/bioinf/plant-multi/#) [63] was used to predict their subcellular localization. The online site psRNATarget (http://plantgrn.noble.org/psRNATarget/analysis) was used to identify the target sites of miRNAs in the *NAC* genes of moso bamboo.

The exon–intron structures were constructed using Gene Structure Display Server (GSDS: http://gsds.cbi.pku.edu.cn/) [64] according to the obtained coding sequence (CDS) and genomic sequences of the *PeNAC*s. In addition, the conserved motifs of PeNACs were identified through the MEME online tool (multiple expectation maximization for motif elicitation, version 4.11.0, http://meme-suite.org/tools/meme) [65] with the default parameters, and the maximum number of motifs was set to 8 with a minimum width of six and a maximum width of 250 amino acids.

### 4.3. Phylogenetic Analysis and Functional Prediction

Both PeNACs and AtNACs were used for phylogenetic analysis, and the sequences of AtNACs were downloaded from the *Arabidopsis* genome site TAIR (The Arabidopsis Information Resource) release 10.0 (http://www.arabidopsis.org/). To explore the evolutionary relationships of NACs between moso bamboo and *Arabidopsis*, Clustal W was used for multiple sequence alignment, and the MEGA 6.0 program (http://www.megasoftware.net/mega.php) was used to construct a neighbor-joining (N-J) phylogenetic tree with the following parameters: N-J tree method, complete deletion, and bootstrap analysis with 1000 replicates [66]. Through the phylogenetic tree described above, the functions of NACs in moso bamboo were predicted based on a homology analysis using the NACs of *Arabidopsis*.

### 4.4. Tissue-Specific Expression Analysis of PeNACs Based on Transcriptome Data

To investigate the expression of the *PeNAC*s in different tissues of moso bamboo, transcriptome data from seven different tissues (leaves, the panicle at the early stage, the panicle at the flowering stage, rhizomes, roots, 20 cm, and 50 cm bamboo shoots) were downloaded from the Short Read Archive of NCBI (Accession number: SRX082501-SRX082512). Matrix2png (https://matrix2png.msl.ubc.ca/) was used to plot a gene expression map to analyze the expression abundance of the *PeNAC*s using RPKM (reads per kilobase per million mapped reads) value. For statistical convenience, logarithm (Log) base 2 was used to analyze the expression.

### 4.5. Co-Expression Network of PeNACs and Subsequent GO Analysis

A co-expression analysis of the *PeNAC*s involved in SCW biosynthesis was performed by using the BambooNET database (http://bioinformatics.cau.edu.cn/bamboo/) [35]. The transcriptional data included rhizome, root, shoot, leaf, sheath, and bud during different development stages. The co-expression relationships between the candidate genes and co-expressed genes were measured. For the candidate genes in the co-expression list, GO analysis was performed to reveal the role of *PeNAC*s in SCW biosynthesis and visualized by TBtools. The subsequent GO analysis was conducted on the same platform, with *p*-values and FDRs (false discovery rates) lower than 0.01 and 0.05, respectively. In addition, the sequences and the expression data of *PeMYB*s were downloaded from PLAZA (https://bioinformatics.psb.ugent.be/plaza/) and BambooNET. The *PeMYB*s positively co-expressed with *PeNAC*s were measured, and the Cytoscape software was used to make the co-expression network [67].

### 4.6. Plant Material Collection and cDNA Synthesis

To validate the functions of *PeNAC*s involved in the regulation of the SCW in moso bamboo, shoots were gathered in Nanchang City, Jiangxi Province, China (E 115°46′1″; N 28°45′57″). For gene expression pattern analysis, the basal parts of the 13th internode were collected from shoots of different heights (1.0 m, 2.0 m, 4.0 m, 6.0 m, and 8.0 m), which represented the shoots at different developmental stages. Each sample included at least three individuals. The collected samples were quickly frozen in liquid nitrogen and stored at −80 °C for the further extraction of RNA; meanwhile, some of the samples were stored in formalin-acetic acid-alcohol (FAA) at 4 °C for sectioning.

Total RNA from the shoots was isolated using TRIzol reagent solution (Invitrogen, USA) following the manufacturer’s protocol. At least three biological replicates were performed for all samples. Recombinant DNase I (Takara, Japan) was used to remove residual DNA from each sample. The integrity of the total RNA was verified through agarose gel electrophoresis, and the purity and concentration of the total RNA were determined by spectrophotometry (Nanodrop 2000). First-strand cDNA was synthesized using a reverse transcription system (Promega, USA). For each 20 μL reaction, 1000 ng of total RNA was used, and the synthesis was carried out at 42 °C for 45 min and 99 °C for 5 min, followed by 4 °C storage. The final cDNA product was diluted five-fold prior to use.

### 4.7. Real-Time Quantitative PCR Analysis

*PeNAC*s associated with SCW biosynthesis were screened according to the phylogenetic relationships between PeNACs and AtNACs. Based on the multiple alignments of the *PeNAC*s and the positively co-expressed *PeMYB*s, the specific primers for analyzing the expression patterns of the *PeNAC*s and *PeMYB*s [29] were designed using Primer Premier 5.0 software. The expression of miR164 targeted *PeNAC*s was also analyzed using the primers [35]. The primers were synthesized by Shanghai Sangon Biotech, and the primer information was shown in Table S5.

The qRT-PCR experiments were performed on a qTOWER2.2 system (Analytik Jena, Germany) with the Roche Light Cycler^®^ 480 SYBR Green 1 Master kit (Roche, USA). The reaction volume was 10.0 μL, containing 5.0 μL 2× SYBR Green 1 Master, 0.8 μL cDNA, 0.2 μL of each primer (10 μM), and 4.0 μL ddH_2_O. The qRT-PCR procedure was as follows: denaturation at 95 °C for 10 min and 40 cycles at 95 °C for 10 s and 60 °C for 10 s. Gene expression was calculated using the 2^−ΔΔCT^ method [68] with the *PeNTB* as the reference gene. The qRT-PCR experiments were performed in biological triplicate with three technical replicates.

### 4.8. Histological Analysis Method

The samples were removed from the FAA fixative, rinsed with deionized water and cut to the appropriate size (~8 mm^3^). Polyethylene glycol (PEG) was used for a series of dehydration reactions at 80 °C. In the first step, the samples were immersed in a mixture of PEG 1000 and an equal volume of deionized water, followed by an equal volume of PEG 1000 and PEG 4000, and finally, PEG 4000. The samples were embedded in PEG 4000, and a rotary microtome (Leica RM2165, Germany) was used to make sections. The sections (10 µm) were prepared and gently placed in a petri dish containing deionized water, followed by staining with a drop of 0.5% toluidine blue at 80 °C for 3 min [69]. The sections were washed with deionized water. Cover glasses were placed on the stained sections, and nail polish was used to seal the cover glass. Finally, an Olympus CX31 microscope was used to observe and take photos.

### 4.9. Transcriptional Activity Analysis of PeNACs

To verify the transcriptional activity of *PeNAC*s, the coding sequences (CDSs) of three *PeNAC*s associated with SCW biosynthesis were PCR amplified and inserted into the pGBKT7 vectors using the specific primers listed in Table S6. Subsequently, the positive control pGBKT7-53+pGADT7-T, negative control pGBKT7 empty plasmid and pGBKT7-*PeNAC* fusion vectors were transformed into the yeast strain AH109 using the lithium acetate method. The transformed yeast cells were further cultured on SD/-Trp and SD/-Trp/-His/-Ade/X-α-Gal selective media and incubated at 29 °C for 2–3 days.

### 4.10. Statistical Analysis

IBM SPSS Statistics (Version 21.0) was used for statistical analysis, and the mean and standard deviations of three biological replicates are presented. Significant differences are indicated at * *p* < 0.05 and ** *p* < 0.01.

## 5. Conclusions

At present, bamboo is a good wood substitute in China. NAC TFs can regulate SCW biosynthesis and lignification deposition, which affect the wood properties of bamboo. Therefore, the study of bamboo NAC TFs has great significance for revealing the mechanism of bamboo material production. A total of 94 PeNACs were identified and characterized in moso bamboo, including gene structures, conserved motifs, phylogenetic relationships, and expression patterns. The PeNACs were clustered into 11 subgroups (S1–S11) based on phylogenetic relationships. Gene structure analysis showed that the sizes and relative positions of exons were conserved and diverse. The functions of the PeNACs were predicted according to their phylogenetic relationships with the AtNACs verified in *Arabidopsis*. The expression patterns of 15 *PeNAC*s related to SCW biosynthesis were validated by qRT-PCR, which indicated that with the increasing shoot height, they were all upregulated significantly. Additionally, three *PeNAC*s were verified to be transcriptional activators for the regulation of SCW biosynthesis. This finding provides a starting point for the functional study of *PeNAC*s associated with SCW biosynthesis, further research is still required to define the biological functions and molecular mechanisms of the *PeNAC*s in bamboo.

## Supplementary Materials

The following are available online at https://www.mdpi.com/2218-273X/9/10/609/s1. Table S1: The basic physical and chemical characteristics, and subcellular localization of proteins encoded by *PeNAC*s in moso bamboo, Table S2: NAC proteins from moso bamboo and Arabidopsis, Table S3: Different *PeNAC*s targeted by the same miRNA, Table S4: Different types of miRNAs found in the same *PeNAC*, Table S5: Specific primers of *PeNAC*s, miR164 and *PeMYB*s for qRT-PCR, Table S6: Specific primers of *PeNAC*s for transcriptional activation activity experiment, Figure S1: Expression analysis of 3 *PeNAC*s and *miR164* in different height shoots using qRT-PCR. *PeNTB* was used as the reference gene. Average and error bars represented standard deviation of three biological replicates. Asterisks indicated significant difference compared to the transcription level of control groups (**p* < 0.05, and ***p* < 0.01). 1: 1.0 m shoots; 2: 2.0 m shoots; 3: 4.0 m shoots; 4: 6.0 m shoots; 5: 8.0 m shoots, Figure S2: Phylogenetic tree based on the proteins of moso bamboo and Arabidopsis. The tree was constructed using the N-J method with 1000 bootstrap replicates as implemented in MEGA6.0.
