# Supplementary material for: Genome-Wide Investigation of the NAC Gene Family and Its Potential Association with the Secondary Cell Wall in Moso Bamboo
Source: Biomolecules. 2019 Oct 14;9(10):609. doi: 10.3390/biom9100609 (PMC6843218; doi:10.3390/biom9100609)
Supplement: Supplementary file 1 [file biomolecules-09-00609-s001.zip › Supplementary files/Figure S1.docx]

**Figure S1. Expression analysis of 3 *PeNAC*s and *miR164* in different height shoots using qRT-PCR.** *PeNTB* was used as the reference gene. Average and error bars represented standard deviation of three biological replicates. Asterisks indicated significant difference compared to the transcription level of control groups (**p* < 0.05, ***p* < 0.01). 1: 1.0 m shoots; 2: 2.0 m shoots; 3: 4.0 m shoots; 4: 6.0 m shoots; 5: 8.0 m shoots.
