# Supplementary material for: Genome-Wide Investigation of the NAC Gene Family and Its Potential Association with the Secondary Cell Wall in Moso Bamboo
Source: Biomolecules. 2019 Oct 14;9(10):609. doi: 10.3390/biom9100609 (PMC6843218; doi:10.3390/biom9100609)
Supplement: Supplementary file 1 [file biomolecules-09-00609-s001.zip › Supplementary files/Figure S2.docx]

**Figure S2. Phylogenetic tree based on the proteins of moso bamboo and Arabidopsis.** The tree was constructed using the N-J method with 1000 bootstrap replicates as implemented in MEGA6.0.
