# Supplementary material for: Genome-Wide Investigation of the NAC Gene Family and Its Potential Association with the Secondary Cell Wall in Moso Bamboo
Source: Biomolecules. 2019 Oct 14;9(10):609. doi: 10.3390/biom9100609 (PMC6843218; doi:10.3390/biom9100609)
Supplement: Supplementary file 1 [file biomolecules-09-00609-s001.zip › Supplementary files/Table S1.docx]

**Table S1. The basic physical and chemical characteristics, and subcellular localization of proteins encoded by *PeNAC*s in moso bamboo.**

| **Nomenclature** | **Bamboo GDB assembly name** | **Basic feature of protein** | | | **Subcellular location** |
| --- | --- | --- | --- | --- | --- |
|  |  | **L (aa)** | **MW (Da)** | **pI** |  |
| *PeNAC01* | PH01000001G1600 | 317 | 36597.23 | 6.15 | Nucleus |
| *PeNAC02* | PH01000002G2670 | 250 | 26883.67 | 8.98 | Nucleus |
| *PeNAC03* | PH01000003G1230 | 390 | 41918.56 | 6.31 | Nucleus |
| *PeNAC04* | PH01000009G0870 | 283 | 32109.34 | 5.55 | Nucleus |
| *PeNAC05* | PH01000017G0480 | 646 | 72255.86 | 5.76 | Nucleus |
| *PeNAC06* | PH01000021G0720 | 640 | 70341.62 | 4.56 | Nucleus |
| *PeNAC07* | PH01000041G2170 | 340 | 37228.14 | 6.67 | Nucleus |
| *PeNAC08* | PH01000044G0380 | 284 | 32302.96 | 6.07 | Nucleus |
| *PeNAC09* | PH01000053G1640 | 361 | 39491.16 | 8.17 | Nucleus |
| *PeNAC10* | PH01000053G1650 | 361 | 40385.36 | 6.32 | Nucleus |
| *PeNAC11* | PH01000059G0340 | 338 | 37810.29 | 5.11 | Nucleus |
| *PeNAC12* | PH01000063G0560 | 249 | 27052.44 | 9.69 | Nucleus |
| *PeNAC13* | PH01000070G0710 | 623 | 67787.79 | 4.58 | Nucleus |
| *PeNAC14* | PH01000070G1490 | 348 | 38650.00 | 9.33 | Nucleus |
| *PeNAC15* | PH01000071G0980 | 271 | 30932.90 | 10.85 | Nucleus |
| *PeNAC16* | PH01000077G1100 | 366 | 40879.94 | 5.82 | Nucleus |
| *PeNAC17* | PH01000077G1110 | 388 | 41669.53 | 8.20 | Nucleus |
| *PeNAC18* | PH01000093G0340 | 289 | 32501.63 | 8.18 | Nucleus |
| *PeNAC19* | PH01000110G0680 | 316 | 34476.21 | 9.08 | Nucleus |
| *PeNAC20* | PH01000111G0400 | 450 | 49667.65 | 4.62 | Nucleus |
| *PeNAC21* | PH01000111G0850 | 300 | 32814.51 | 8.87 | Nucleus |
| *PeNAC22* | PH01000117G0520 | 255 | 27885.78 | 9.93 | Nucleus |
| *PeNAC23* | PH01000122G1000 | 526 | 58151.27 | 8.07 | Nucleus |
| *PeNAC24* | PH01000140G1040 | 665 | 72896.07 | 4.58 | Nucleus |
| *PeNAC25* | PH01000141G0120 | 297 | 32709.28 | 8.74 | Nucleus |
| *PeNAC26* | PH01000141G1130 | 381 | 41601.58 | 9.56 | Nucleus |
| *PeNAC27* | PH01000148G1280 | 267 | 29824.96 | 5.60 | Nucleus |
| *PeNAC28* | PH01000177G0520 | 405 | 45370.22 | 8.19 | Nucleus |
| *PeNAC29* | PH01000183G1320 | 318 | 34866.82 | 9.18 | Nucleus |
| *PeNAC30* | PH01000260G0100 | 344 | 37552.54 | 7.08 | Nucleus |
| *PeNAC31* | PH01000261G0890 | 245 | 27143.44 | 8.57 | Nucleus |
| *PeNAC32* | PH01000298G0850 | 308 | 33733.96 | 8.98 | Nucleus |
| *PeNAC33* | PH01000309G0360 | 256 | 27831.91 | 5.56 | Nucleus |
| *PeNAC34* | PH01000317G0680 | 558 | 62084.58 | 4.64 | Nucleus |
| *PeNAC35* | PH01000331G1100 | 317 | 35890.66 | 6.20 | Nucleus |
| *PeNAC36* | PH01000352G0610 | 372 | 40613.51 | 6.56 | Nucleus |
| *PeNAC37* | PH01000358G0570 | 556 | 62925.87 | 9.02 | Nucleus |
| *PeNAC38* | PH01000376G0720 | 333 | 37604.45 | 7.62 | Nucleus |
| *PeNAC39* | PH01000381G0780 | 389 | 43603.71 | 5.43 | Nucleus |
| *PeNAC40* | PH01000382G0290 | 469 | 51802.30 | 4.64 | Nucleus |
| *PeNAC41* | PH01000383G0210 | 694 | 76916.46 | 4.93 | Nucleus |
| *PeNAC42* | PH01000439G0460 | 375 | 40949.88 | 6.56 | Nucleus |
| *PeNAC43* | PH01000468G0060 | 354 | 39038.10 | 5.81 | Nucleus |
| *PeNAC44* | PH01000483G1000 | 292 | 32896.26 | 6.96 | Nucleus |
| *PeNAC45* | PH01000491G0390 | 435 | 48646.27 | 9.23 | Nucleus |
| *PeNAC46* | PH01000501G0450 | 355 | 38704.79 | 6.92 | Nucleus |
| *PeNAC47* | PH01000589G0830 | 325 | 36240.48 | 5.28 | Nucleus |
| *PeNAC48* | PH01000652G0840 | 353 | 38941.77 | 5.83 | Nucleus |
| *PeNAC49* | PH01000653G0110 | 217 | 25201.17 | 8.19 | Nucleus |
| *PeNAC50* | PH01000667G0650 | 316 | 34047.11 | 5.38 | Nucleus |
| *PeNAC51* | PH01000724G0330 | 313 | 34570.11 | 7.10 | Nucleus |
| *PeNAC52* | PH01000729G0360 | 380 | 42554.81 | 6.54 | Nucleus |
| *PeNAC53* | PH01000762G0770 | 292 | 32936.13 | 7.75 | Nucleus |
| *PeNAC54* | PH01000794G0630 | 308 | 33969.58 | 8.79 | Nucleus |
| *PeNAC55* | PH01000832G0670 | 650 | 71413.19 | 5.20 | Nucleus |
| *PeNAC56* | PH01000845G0490 | 290 | 33073.78 | 6.01 | Nucleus |
| *PeNAC57* | PH01000856G0430 | 395 | 42486.48 | 6.46 | Nucleus |
| *PeNAC58* | PH01000891G0360 | 317 | 34680.14 | 6.14 | Nucleus |
| *PeNAC59* | PH01000970G0550 | 662 | 72195.65 | 4.72 | Nucleus |
| *PeNAC60* | PH01001063G0490 | 640 | 71208.66 | 4.69 | Nucleus |
| *PeNAC61* | PH01001153G0250 | 364 | 39396.43 | 6.66 | Nucleus |
| *PeNAC62* | PH01001168G0540 | 364 | 40366.62 | 9.31 | Nucleus |
| *PeNAC63* | PH01001177G0140 | 317 | 35237.79 | 7.76 | Nucleus |
| *PeNAC64* | PH01001184G0040 | 333 | 36646.81 | 5.94 | Nucleus |
| *PeNAC65* | PH01001195G0450 | 313 | 35085.89 | 7.80 | Nucleus |
| *PeNAC66* | PH01001292G0350 | 358 | 38855.80 | 8.84 | Nucleus |
| *PeNAC67* | PH01001309G0120 | 293 | 33101.46 | 7.63 | Nucleus |
| *PeNAC68* | PH01001428G0090 | 322 | 35494.71 | 6.20 | Nucleus |
| *PeNAC69* | PH01001618G0390 | 292 | 32630.59 | 6.65 | Nucleus |
| *PeNAC70* | PH01001652G0230 | 653 | 71089.70 | 4.58 | Nucleus |
| *PeNAC71* | PH01001664G0230 | 456 | 50426.89 | 4.66 | Nucleus |
| *PeNAC72* | PH01001683G0170 | 357 | 39405.11 | 6.31 | Nucleus |
| *PeNAC73* | PH01001753G0040 | 317 | 34714.90 | 9.04 | Nucleus |
| *PeNAC74* | PH01001770G0220 | 281 | 31260.64 | 9.00 | Nucleus |
| *PeNAC75* | PH01001797G0410 | 577 | 64095.44 | 5.16 | Nucleus |
| *PeNAC76* | PH01001896G0060 | 254 | 28096.50 | 8.89 | Nucleus |
| *PeNAC77* | PH01002026G0020 | 650 | 70788.70 | 4.69 | Nucleus |
| *PeNAC78* | PH01002131G0260 | 251 | 27484.29 | 10.07 | Nucleus |
| *PeNAC79* | PH01002245G0380 | 403 | 42967.03 | 4.55 | Nucleus |
| *PeNAC80* | PH01002276G0270 | 345 | 37493.42 | 6.89 | Nucleus |
| *PeNAC81* | PH01002611G0200 | 400 | 45184.04 | 9.06 | Nucleus |
| *PeNAC82* | PH01002631G0130 | 241 | 26755.35 | 8.79 | Nucleus |
| *PeNAC83* | PH01002777G0100 | 269 | 29608.93 | 8.71 | Nucleus |
| *PeNAC84* | PH01002998G0240 | 425 | 46721.69 | 4.61 | Nucleus |
| *PeNAC85* | PH01003084G0080 | 365 | 41975.05 | 6.33 | Nucleus |
| *PeNAC86* | PH01003138G0210 | 265 | 29150.91 | 9.01 | Nucleus |
| *PeNAC87* | PH01003190G0100 | 321 | 35174.69 | 6.32 | Nucleus |
| *PeNAC88* | PH01003898G0190 | 389 | 43678.14 | 5.43 | Nucleus |
| *PeNAC89* | PH01004006G0090 | 335 | 37391.77 | 8.07 | Nucleus |
| *PeNAC90* | PH01004129G0090 | 469 | 52360.60 | 9.40 | Nucleus |
| *PeNAC91* | PH01004181G0160 | 296 | 33351.75 | 7.68 | Nucleus |
| *PeNAC92* | PH01004390G0110 | 275 | 30663.15 | 6.36 | Nucleus |
| *PeNAC93* | PH01004605G0030 | 510 | 56309.77 | 5.23 | Nucleus |
| *PeNAC94* | PH01006140G0010 | 317 | 34492.67 | 8.77 | Nucleus |

Note: L, MW and pI indicated protein length, molecular weight and theoretical isoelectric point, respectively.
