# Supplementary material for: Genome-Wide Investigation of the NAC Gene Family and Its Potential Association with the Secondary Cell Wall in Moso Bamboo
Source: Biomolecules. 2019 Oct 14;9(10):609. doi: 10.3390/biom9100609 (PMC6843218; doi:10.3390/biom9100609)
Supplement: Supplementary file 1 [file biomolecules-09-00609-s001.zip › Supplementary files/Table S5.docx]

**Table S5. Specific primers of *PeNAC*s, *miR164* and *PeMYB*s for qRT-PCR.**

| **Gene name** | **Primer sequence** |
| --- | --- |
| *PeNAC1* | F: 5′-CGCTTGGAGACCAACGAA-3′  R: 5′-ACGCGACATGGTCGTTGA-3′ |
| *PeNAC3* | F: 5′-CAAAAGTCCGATTGGATCATGCA-3′  R: 5′-GTGGTGCTTCTTCTTGAACACCCT-3′ |
| *PeNAC8* | F: 5′-CGCATTTCAGAAGCCGAC-3′  R: 5′-GTGAGCCCAAGAAATGGTG-3′ |
| *PeNAC11* | F: 5′-GTCGGCTACTACCTCGTGAA-3′  R: 5′-CCGGTCCTTGTAGCTGAAGA-3′ |
| *PeNAC32* | F: 5′-AACTCCGTTCACGGTGTG-3′  R: 5′-TGCCCTGTTAGTTGGGAC-3′ |
| *PeNAC36* | F: 5′-GAGTACCGCCTCGACGACATG-3′  F: 5′-GATCACCCACCCTTCCTCCTG-3′ |
| *PeNAC37* | F: 5′-TCTCGATCATGATCAGTTCCA-3′  R: 5′-GCTGTTGATGCTGGTGCT-3′ |
| *PeNAC42* | F: 5′-GTGCAAGCAGGAGACCAA-3′  R: 5′-AGTACTCGGCTGGGTTCG-3′ |
| *PeNAC45* | F: 5′-CTGACCAACAGTAAAAATGGTGGA-3′  R: 5′-GCCGAGGTGATACTGGTGAA-3′ |
| *PeNAC56* | F: 5′-CAAGTAAAGAGGAAGGCTGGGTG-3′  R: 5′-GTGCGTTTGGCAGGTCGTAGT-3′ |
| *PeNAC73* | F: 5′-AGCAAGGACGCCGTCC-3′  R: 5′-AACCGCCCTGTTGTTAGG-3′ |
| *PeNAC76* | F: 5′-GCGGACTCCTCGCTGCAGTA-3′  R: 5′-GCTCGTGCTCCTGCTTGCAC-3′ |
| *PeNAC81* | F: 5′-GCAGCATTTGAAACATGTGT-3′  R: 5′-GCTGATCGGATGACACCT-3′ |
| *PeNAC85* | F: 5′-GTAGGATAGGGCTCTCCGAGT-3′  R: 5′-TGCGCTTTGCATAGATGG-3′ |
| *PeNAC94* | F: 5′-TCGTCGCGGGAAACAAC-3′  R: 5′-GCTGCTGGGCTGTAGAAAT-3′ |
| *miR164* | F: 5′-AGCAGCATTGGAGAAGCAGGG-3′  R: 5′-CTCAACTGGTGTCGTGGAGTC-3′ |
| *PeMYB3* | F: 5′-GCAGTGTGCTCTATGGTGTGC-3′  R: 5′-ATGTGCTGTTGGTGACCTGC-3′ |
| *PeMYB14* | F: 5′-CGGAGTCGAGCACGAACA-3′  R: 5′-GCGATGCTGCTGAACTCG-3′ |
| *PeMYB26* | F: 5′-TGATTGGGACCAGGGCAAA-3′  R: 5′-CCTCCCTCTTCACAGGCTTCAT-3′ |
| *PeMYB35* | F: 5′-GGACTGCGTAGGGGAGGA-3′  R: 5′-CCTGCTGCTTCTGCTCGTA-3′ |
| *PeMYB37* | F: 5′-CCATGCCTGCTACGAGCAT-3′  R: 5′-TGGTGTTAGTTCTTGATCCCACAT-3′ |
| *PeMYB50* | F: 5′-CGTCATCGCCAGGCTTTT-3′  R: 5′-ACAGCCTCATCCGCTCCCT-3′ |
| *PeMYB68* | F: 5′-CTCCCCGGTGAGCAGCTT-3′  R: 5′-GCCGGTGTAGGCGCTGTA-3′ |
| *PeNTB* | F: 5′-TCTTGTTTGACACCGAAGAGGAG-3′  R: 5′-AATAGCTGTCCCTGGAGGAGTTT-3′ |
