# Supplementary material for: Genome-Wide Investigation of the NAC Gene Family and Its Potential Association with the Secondary Cell Wall in Moso Bamboo
Source: Biomolecules. 2019 Oct 14;9(10):609. doi: 10.3390/biom9100609 (PMC6843218; doi:10.3390/biom9100609)
Supplement: Supplementary file 1 [file biomolecules-09-00609-s001.zip › Supplementary files/Table S6.docx]

**Table S6. Specific primers of *PeNAC*s for transcriptional activation activity experiment.**

| **Gene name** | **Primer sequence** |
| --- | --- |
| *PeNAC8* | F: 5′-CGGAATTCATGGACCAGGAGG-3′  R: 5′-CGGGATCCTCACTGCAGGGA-3′ |
| *PeNAC36* | F: 5′-CGGAATTCATGAGCATCTCGG-3′  R: 5′-CGGGATCCTTATGCGTTATTCAT-3′ |
| *PeNAC73* | F: 5′-CGGAATTCATGACATGGTGCAA-3′  R: 5′-CGGGATCCTCAGGGACCAAA-3′ |
